# Crystal structure of bis­[1-(2-hy­droxy­eth­yl)-2-methyl-5-nitro-1*H*-imidazole-κ*N* ^3^]silver(I) tetra­fluorido­borate methanol monosolvate

**DOI:** 10.1107/S2056989015002819

**Published:** 2015-02-21

**Authors:** Joshua H. Palmer, Rita K. Upmacis

**Affiliations:** a Department of Chemistry, Columbia University, New York, NY 10027, USA; b Haskins Laboratories, Dept. of Chemistry, Pace University, New York, NY 10038, USA

**Keywords:** crystal structure, silver, metronidazole, Flag­yl, tetra­fluorido­borate, hydrogen bonding

## Abstract

1-(2-Hy­droxy­eth­yl)-2-methyl-5-nitro-1*H*-imidazole (metronidazole, MET) is a medication that is used to treat infections by a variety of anaerobic organisms, but there are relatively few reports of the structures of metal compounds that exhibit coordination of metronidazole. We have demonstrated that MET reacts with AgBF_4_ to give [Ag(MET)_2_]BF_4_·CH_3_OH, in which the Ag^I^ cation is coordinated by two MET ligands with a *trans* arrangement. The structure of [Ag(MET)_2_]BF_4_ exhibits some inter­esting differences from its nitrate counterpart, [Ag(MET)_2_]NO_3_ [Fun *et al.* (2008). *Acta Cryst.* E**64**, m668]. For instance, although the two MET ligands of both [Ag(MET)_2_]BF_4_ and [Ag(MET)_2_]NO_3_ are almost coplanar, the former compound has an *anti*-like geometry with a mol­ecular inversion center, but the latter has a *syn*-like arrangement. In the crystal, the BF_4_^−^ anion is linked by an O—H⋯F hydrogen bond to the methanol solvent molecule, which is, in turn, linked to the cation by an O—H⋯O hydrogen bond; the components of the structure are linked by O—H⋯O hydrogen bonds, forming chains along [001]. One of the MET ligands and the BF_4_^−^ anion are disordered over two sets of sites with ratios of refined occupancies 0.501 (17):0.499 (17) and 0.539 (19):0.461 (19), respectively.

## Abstract 2

1-(2-Hy­droxy­eth­yl)-2-methyl-5-nitro-1*H*-imidazole (metronidazole, MET) reacts with AgBF_4_ to give [Ag(MET)_2_]BF_4_, in which the Ag atom is coordinated by two MET ligands with a *trans* arrangement.

## Chemical context   

1-(2-Hy­droxy­eth­yl)-2-methyl-5-nitro-1*H*-imidazole, also known as metronidazole (MET) or Flagyl, is a medication used particularly for treatment of parasitic infections, such as trichomoniasis, amoebiasis and giardiasis, but is also effective against anaerobic bacteria (Freeman *et al.*, 1997 ▸; Miljkovic *et al.*, 2014 ▸; Soares *et al.*, 2012 ▸; Samuelson, 1999 ▸; Lofmark *et al.*, 2010 ▸). There are relatively few reports of the structures of metal compounds that exhibit coordination of MET. For example, with respect to silver, only the nitrate compound, [Ag(MET)_2_]NO_3_, has been structurally characterized by X-ray diffraction (Fun *et al.*, 2008 ▸). Herein, we describe the structure of the tetra­fluorido­borate derivative, [Ag(MET)_2_]BF_4_, which is obtained by addition of MET to AgBF_4_ in methanol (see Scheme).

## Structural commentary   

**Figure 1:**
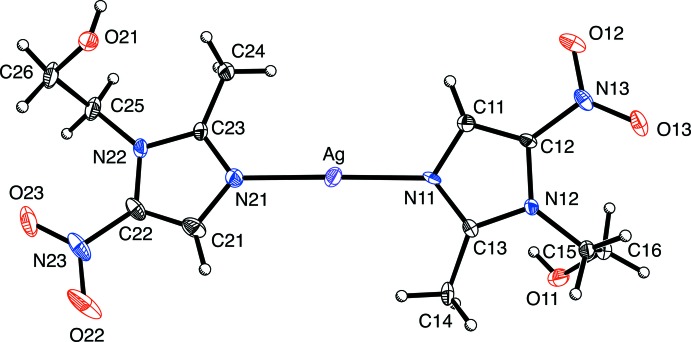
The mol­ecular structure of the cation of the title compound, with displacement ellipsoids drawn at the 30% probability level. The disorder is not shown.

Crystals of composition [Ag(MET)_2_]BF_4_·MeOH were obtained from a solution in methanol. The asymmetric unit consists of a silver cation, [Ag(MET)_2_]^+^, a tetra­fluorido­borate anion, BF_4_^−^, and a solvent methanol mol­ecule. The silver atom of [Ag(MET)_2_]^+^ is coordinated by two MET ligands in a *trans* manner by their N^3^ nitro­gen atoms, as illustrated in Fig. 1 ▸.
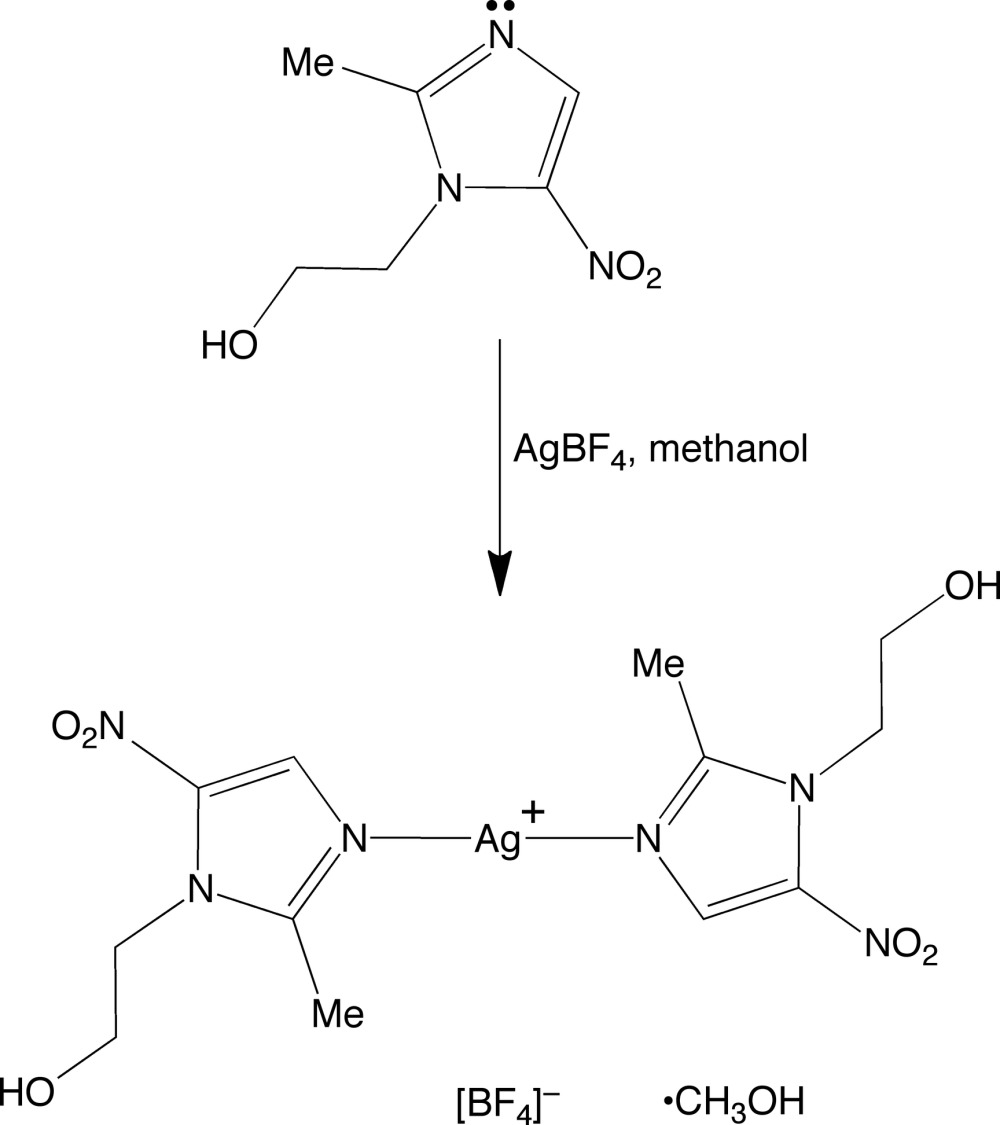


One of the MET ligands exhibits disorder resulting from rotation about the Ag—N bond [the dihedral angle between the planes of the disordered 5-membered rings is 11.0 (9)°]. The Ag—N bond lengths [Ag—N11 = 2.082 (15), Ag—N11*A* = 2.163 (16) and Ag—N21 = 2.1193 (15) Å] are comparable to those values in the nitrate derivative, [2.1489 (11) and 2.1475 (11) Å; Fun *et al.*, 2008 ▸). There are, however, some inter­esting differences between the two compounds.

**Figure 2:**
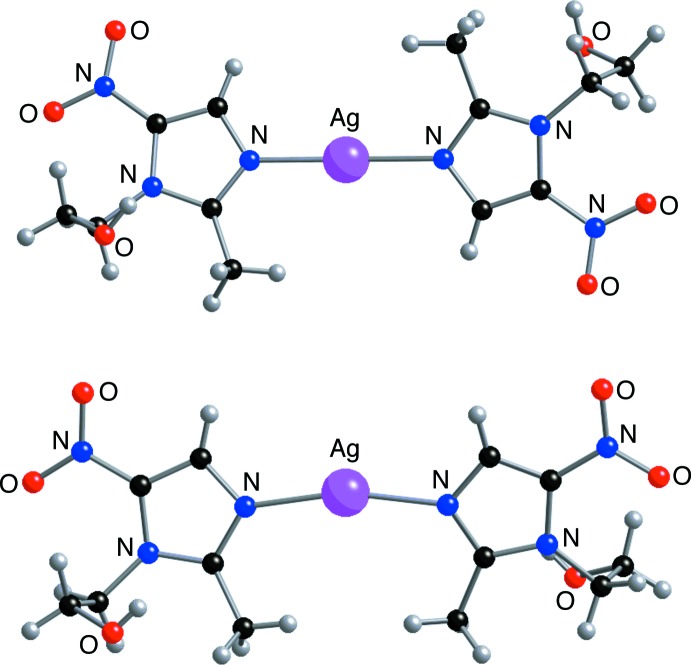
Comparison of the [Ag(MET)_2_]^+^ units in [Ag(MET)_2_]BF_4_ (top) and [Ag(MET)_2_]NO_3_ (bottom).

First, while the two MET ligands of both [Ag(MET)_2_]BF_4_ and [Ag(MET)_2_]NO_3_ are almost coplanar, the former compound has an *anti*-like geometry, and the latter has a *syn*-like arrangement. Thus, the C13—N11⋯N21—C23 torsion angle for [Ag(MET)_2_]BF_4_ is 160.8 (9)° [148.6 (11)° for the minor component of disorder], while the value for [Ag(MET)_2_]NO_3_ is 24.10° (Fun *et al.*, 2008 ▸). These differences are illustrated in Fig. 2 ▸, which shows that the [Ag(MET)_2_]^+^ unit of [Ag(MET)_2_]BF_4_ has an approximate inversion center at the Ag^I^ ion, whereas [Ag(MET)_2_]NO_3_ does not.

A second inter­esting difference is that the N11—Ag—N21 angle of 175.7 (5)° for [Ag(MET)_2_]BF_4_ is much closer to 180° than is the corresponding value for [Ag(MET)_2_]NO_3_ [165.34 (4)°; Fun *et al.*, 2008 ▸). It is possible that this could be attributed to the tetra­fluorido­borate ligand being considered a non-coordinating ion relative to nitrate, and this is reflected by the fact that [Ag(MET)_2_]NO_3_ exhibits Ag⋯O contacts of 2.63 and 2.67 Å, which are comparable to distances in other silver nitrate compounds (Wu *et al.*, 2012 ▸).

## Supra­molecular features   

**Figure 3:**
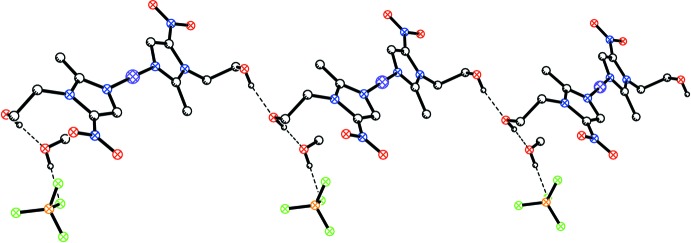
Part of a hydrogen-bonded chain along [001]. The disorder is not shown and hydrogen bonds are shown as dashed lines.

The hy­droxy­ethyl group of one of the MET ligands [O21—H] serves as a donor in an inter­molecular hydrogen-bonding inter­action with the other hy­droxy­ethyl group [O11—H] of an adjacent mol­ecule. In turn, the latter hy­droxy­ethyl group serves as a hydrogen-bond donor to a methanol mol­ecule, which also hydrogen bonds to a tetra­fluorido­borate anion. In the crystal, the components of the structure are linked into chains along [001] by the O—H⋯O hydrogen bonds (Table 1 ▸ and Fig. 3 ▸).

## Database survey   

In addition to coordination to silver, metronidazole has also been shown to coordinate to other metals, and structurally characterized compounds have been reported for Co (Galván-Tejada *et al.*, 2002 ▸), Cu (Galván-Tejada *et al.*, 2002 ▸; Barba-Behrens *et al.*, 1991 ▸; Athar *et al.*, 2005 ▸; Ratajczak-Sitarz *et al.*, 1998 ▸; Bharti *et al.*, 2002 ▸), Zn (Galván-Tejada *et al.*, 2002 ▸), Ru (Wu *et al.*, 2003 ▸; Kennedy *et al.*, 2006 ▸), Rh (Dyson *et al.*, 1990 ▸; Nothenberg *et al.*, 1994 ▸), Pd (Bharti *et al.*, 2002 ▸; De Bondt *et al.*, 1994 ▸; Rochon *et al.*, 1993 ▸) and Pt (Bharti *et al.*, 2002 ▸; Bales *et al.*, 1983 ▸). In these compounds, the coordination number of the central atom ranges from four for Cu, Zn, Pd and Pt to six for Ru and Rh.

## Synthesis and crystallization   

Crystals of composition [Ag(MET)_2_]BF_4_·MeOH were obtained by combining AgBF_4_ with MET in a 1:2 molar ratio in methanol and allowing the solution to evaporate slowly at room temperature.

## Refinement   

Crystal data, data collection and structure refinement details are summarized in Table 2 ▸. Hydrogen atoms were refined using a riding-model approximation with C—H = 0.95–0.99 Å, O—H = 0.84 Å and *U*_iso_(H) = 1.2*U*_eq_(C,O). One of the MET ligands was refined as rotationally disordered with occupancies of 0.501 (17) and 0.499 (17) and the configurations were modeled using the SAME command in *SHELXL2013* (Sheldrick, 2015 ▸). The tetra­fluorido­borate counter-ion was also refined as disordered and was modeled with two site occupancies, 0.539 (19) and 0.461 (19).

## Supplementary Material

Crystal structure: contains datablock(s) I. DOI: 10.1107/S2056989015002819/lh5749sup1.cif

Structure factors: contains datablock(s) I. DOI: 10.1107/S2056989015002819/lh5749Isup2.hkl

Supporting information file. DOI: 10.1107/S2056989015002819/lh5749Isup3.mol

Supporting information file. DOI: 10.1107/S2056989015002819/lh5749Isup4.txt

CCDC reference: 1048516

Additional supporting information:  crystallographic information; 3D view; checkCIF report

## Figures and Tables

**Table 1:** Hydrogen-bond geometry (, )

*D*H*A*	*D*H	H*A*	*D* *A*	*D*H*A*
O1H1F2	0.84	1.84	2.673(9)	173
O11H11*A*O1	0.84	1.86	2.697(10)	175
O21H21*A*O11^i^	0.84	1.91	2.726(11)	164
O21H21*A*O11*A* ^i^	0.84	1.87	2.712(11)	176

Symmetry code: (i) 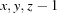.

**Table 2:** Experimental details

Crystal data	
Chemical formula	[Ag(C_6_H_9_N_3_O_3_)_2_]BF_4_CH_4_O
*M*_r_	569.04
Crystal system, space group	Triclinic, *P* 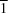
Temperature (K)	130
*a*, *b*, *c* ()	9.2592(10), 10.5339(10), 12.3995(12)
, , ()	106.940(11), 92.788(9), 112.439(10)
*V* (^3^)	1051.7(2)
*Z*	2
Radiation type	Mo *K*
(mm^1^)	1.04
Crystal size (mm)	1.00 0.51 0.31

Data collection	
Diffractometer	Bruker APEXII CCD
Absorption correction	Multi-scan (*SADABS*; Bruker, 2013 ▸)
*T*_min_, *T*_max_	0.551, 0.747
No. of measured, independent and observed [*I* > 2(*I*)] reflections	17164, 6401, 6107
*R*_int_	0.023
(sin /)_max_ (^1^)	0.714

Refinement	
*R*[*F* ^2^ > 2(*F* ^2^)], *wR*(*F* ^2^), *S*	0.028, 0.067, 1.12
No. of reflections	6401
No. of parameters	440
No. of restraints	144
H-atom treatment	H-atom parameters constrained
_max_, _min_ (e ^3^)	1.00, 1.03

Computer programs: *APEX2* and *SAINT* (Bruker, 2013 ▸), *SHELXS97* and *SHELXTL* (Sheldrick, 2008 ▸), *SHELXL2013* (Sheldrick, 2015 ▸).

## supplementary crystallographic information

### Crystal data

**Table:**

[Ag(C_6_H_9_N_3_O_3_)_2_](BF_4_)·CH_4_O	*Z* = 2
*M**_r_* = 569.04	*F*(000) = 572
Triclinic, *P*1	*D*_x_ = 1.797 Mg m^−^^3^
*a* = 9.2592 (10) Å	Mo *K*α radiation, λ = 0.71073 Å
*b* = 10.5339 (10) Å	Cell parameters from 9065 reflections
*c* = 12.3995 (12) Å	θ = 2.3–32.9°
α = 106.940 (11)°	µ = 1.04 mm^−^^1^
β = 92.788 (9)°	*T* = 130 K
γ = 112.439 (10)°	Block, colourless
*V* = 1051.7 (2) Å^3^	1.00 × 0.51 × 0.31 mm

### Data collection

**Table:**

Bruker APEXII CCD diffractometer	6107 reflections with *I* > 2σ(*I*)
φ and ω scans	*R*_int_ = 0.023
Absorption correction: multi-scan (*SADABS*; Bruker, 2013)	θ_max_ = 30.5°, θ_min_ = 1.8°
*T*_min_ = 0.551, *T*_max_ = 0.747	*h* = −13→13
17164 measured reflections	*k* = −15→15
6401 independent reflections	*l* = −17→17

### Refinement

**Table:**

Refinement on *F*^2^	Hydrogen site location: inferred from neighbouring sites
Least-squares matrix: full	H-atom parameters constrained
*R*[*F*^2^ > 2σ(*F*^2^)] = 0.028	*w* = 1/[σ^2^(*F*_o_^2^) + (0.0161*P*)^2^ + 1.0163*P*] where *P* = (*F*_o_^2^ + 2*F*_c_^2^)/3
*wR*(*F*^2^) = 0.067	(Δ/σ)_max_ = 0.007
*S* = 1.12	Δρ_max_ = 1.00 e Å^−^^3^
6401 reflections	Δρ_min_ = −1.03 e Å^−^^3^
440 parameters	Extinction correction: *SHELXL2013* (Sheldrick, 2015), Fc^*^=kFc[1+0.001xFc^2^λ^3^/sin(2θ)]^-1/4^
144 restraints	Extinction coefficient: 0.0027 (4)

### Special details

**Table:**

Geometry. All e.s.d.'s (except the e.s.d. in the dihedral angle between two l.s. planes) are estimated using the full covariance matrix. The cell e.s.d.'s are taken into account individually in the estimation of e.s.d.'s in distances, angles and torsion angles; correlations between e.s.d.'s in cell parameters are only used when they are defined by crystal symmetry. An approximate (isotropic) treatment of cell e.s.d.'s is used for estimating e.s.d.'s involving l.s. planes.

### Fractional atomic coordinates and isotropic or equivalent isotropic displacement parameters (Å^2^)

**Table:**

	*x*	*y*	*z*	*U*_iso_*/*U*_eq_	Occ. (<1)
Ag	0.34662 (2)	0.82628 (2)	−0.00390 (2)	0.02854 (5)	
N11	0.4710 (18)	0.786 (2)	0.1171 (7)	0.018 (2)	0.501 (17)
N12	0.5696 (15)	0.7754 (15)	0.2748 (8)	0.0211 (16)	0.501 (17)
N13	0.7178 (8)	0.6262 (7)	0.1956 (9)	0.0273 (14)	0.501 (17)
O11	0.3317 (11)	0.6459 (13)	0.4129 (9)	0.0404 (16)	0.501 (17)
H11A	0.2863	0.5926	0.3453	0.048*	0.501 (17)
O12	0.7349 (8)	0.5491 (6)	0.1064 (9)	0.0406 (13)	0.501 (17)
O13	0.7868 (7)	0.6518 (5)	0.2921 (10)	0.0368 (14)	0.501 (17)
C11	0.5471 (13)	0.6968 (12)	0.0851 (8)	0.0227 (15)	0.501 (17)
H11B	0.5535	0.6475	0.0092	0.027*	0.501 (17)
C12	0.6111 (12)	0.6925 (11)	0.1829 (8)	0.0197 (15)	0.501 (17)
C13	0.4855 (12)	0.8352 (11)	0.2301 (9)	0.0149 (15)	0.501 (17)
C14	0.3914 (18)	0.9109 (16)	0.2896 (10)	0.039 (4)	0.501 (17)
H14A	0.3477	0.9470	0.2375	0.047*	0.501 (17)
H14B	0.4599	0.9932	0.3572	0.047*	0.501 (17)
H14C	0.3043	0.8428	0.3136	0.047*	0.501 (17)
C15	0.5998 (12)	0.7981 (8)	0.3981 (7)	0.0282 (14)	0.501 (17)
H15A	0.5816	0.8841	0.4416	0.034*	0.501 (17)
H15B	0.7124	0.8190	0.4220	0.034*	0.501 (17)
C16	0.4946 (13)	0.6673 (12)	0.4271 (9)	0.0346 (19)	0.501 (17)
H16A	0.5035	0.5792	0.3772	0.042*	0.501 (17)
H16B	0.5316	0.6806	0.5076	0.042*	0.501 (17)
N11A	0.470 (2)	0.778 (2)	0.1207 (9)	0.028 (3)	0.499 (17)
N12A	0.5515 (16)	0.7755 (15)	0.2949 (8)	0.0179 (12)	0.499 (17)
N13A	0.7308 (8)	0.6471 (7)	0.2389 (8)	0.0224 (12)	0.499 (17)
O11A	0.2839 (10)	0.6195 (10)	0.4105 (9)	0.0349 (13)	0.499 (17)
H11C	0.2496	0.5780	0.3398	0.042*	0.499 (17)
O12A	0.7814 (9)	0.5918 (10)	0.1564 (8)	0.0368 (16)	0.499 (17)
O13A	0.7663 (7)	0.6551 (5)	0.3379 (7)	0.0314 (11)	0.499 (17)
C11A	0.5764 (13)	0.7137 (13)	0.1118 (8)	0.0212 (15)	0.499 (17)
H11D	0.6121	0.6788	0.0434	0.025*	0.499 (17)
C12A	0.6218 (12)	0.7076 (11)	0.2150 (7)	0.0170 (13)	0.499 (17)
C13A	0.4578 (12)	0.8126 (11)	0.2331 (8)	0.0140 (15)	0.499 (17)
C14A	0.3830 (17)	0.9117 (14)	0.2874 (9)	0.033 (4)	0.499 (17)
H14D	0.4602	1.0132	0.3073	0.039*	0.499 (17)
H14E	0.3490	0.8932	0.3571	0.039*	0.499 (17)
H14F	0.2906	0.8940	0.2338	0.039*	0.499 (17)
C15A	0.5567 (9)	0.7874 (8)	0.4170 (6)	0.0213 (11)	0.499 (17)
H15C	0.5264	0.8670	0.4571	0.026*	0.499 (17)
H15D	0.6671	0.8140	0.4521	0.026*	0.499 (17)
C16A	0.4482 (11)	0.6486 (10)	0.4345 (9)	0.0275 (16)	0.499 (17)
H16C	0.4661	0.5659	0.3840	0.033*	0.499 (17)
H16D	0.4758	0.6557	0.5148	0.033*	0.499 (17)
N21	0.20394 (19)	0.85591 (17)	−0.12498 (13)	0.0280 (3)	
N22	0.06590 (19)	0.82730 (17)	−0.28744 (13)	0.0270 (3)	
N23	−0.0132 (2)	1.0346 (2)	−0.2186 (2)	0.0433 (5)	
O21	0.25596 (17)	0.86179 (17)	−0.45811 (12)	0.0340 (3)	
H21A	0.2608	0.7842	−0.4975	0.051*	
O22	−0.0234 (2)	1.1214 (2)	−0.1326 (2)	0.0737 (7)	
O23	−0.0629 (3)	1.0220 (3)	−0.3151 (2)	0.0775 (8)	
C21	0.1481 (2)	0.9618 (2)	−0.10391 (19)	0.0350 (4)	
H21B	0.1652	1.0344	−0.0319	0.042*	
C22	0.0644 (2)	0.94554 (19)	−0.20292 (19)	0.0306 (4)	
C23	0.1525 (2)	0.77595 (19)	−0.23615 (14)	0.0250 (3)	
C24	0.1834 (3)	0.6463 (2)	−0.29385 (16)	0.0349 (4)	
H24A	0.2455	0.6290	−0.2379	0.042*	
H24B	0.2429	0.6636	−0.3552	0.042*	
H24C	0.0821	0.5608	−0.3265	0.042*	
C25	−0.0068 (2)	0.7652 (3)	−0.40921 (17)	0.0375 (5)	
H25A	−0.1117	0.7691	−0.4181	0.045*	
H25B	−0.0236	0.6617	−0.4390	0.045*	
C26	0.0986 (3)	0.8488 (3)	−0.4785 (2)	0.0456 (6)	
H26A	0.0535	0.7976	−0.5612	0.055*	
H26B	0.0997	0.9473	−0.4580	0.055*	
C1	0.0949 (3)	0.4974 (3)	0.1155 (3)	0.0598 (7)	
H1A	0.0573	0.5721	0.1520	0.072*	
H1B	0.0045	0.4081	0.0682	0.072*	
H1C	0.1706	0.5328	0.0672	0.072*	
O1	0.1702 (3)	0.4678 (3)	0.20073 (19)	0.0787 (8)	
H1	0.1856	0.3926	0.1706	0.094*	
B1	0.3719 (2)	0.2457 (2)	0.19180 (16)	0.0263 (4)	
F1	0.4132 (11)	0.1347 (10)	0.1303 (10)	0.0303 (13)	0.461 (19)
F2	0.2428 (8)	0.2394 (9)	0.1193 (6)	0.0313 (12)	0.461 (19)
F3	0.4916 (8)	0.3783 (7)	0.2162 (11)	0.0487 (18)	0.461 (19)
F4	0.3177 (7)	0.2190 (9)	0.2882 (5)	0.0395 (15)	0.461 (19)
F1A	0.4089 (12)	0.1282 (10)	0.1456 (10)	0.045 (2)	0.539 (19)
F2A	0.2240 (8)	0.2191 (10)	0.1397 (9)	0.0575 (19)	0.539 (19)
F3A	0.4837 (8)	0.3671 (8)	0.1725 (8)	0.0457 (13)	0.539 (19)
F4A	0.3783 (17)	0.2765 (12)	0.3073 (4)	0.080 (2)	0.539 (19)

### Atomic displacement parameters (Å^2^)

**Table:**

	*U*^11^	*U*^22^	*U*^33^	*U*^12^	*U*^13^	*U*^23^
Ag	0.03598 (8)	0.02979 (7)	0.02076 (7)	0.01318 (6)	0.00356 (5)	0.01063 (5)
N11	0.020 (4)	0.016 (3)	0.025 (4)	0.013 (3)	0.012 (3)	0.008 (3)
N12	0.021 (3)	0.022 (2)	0.020 (3)	0.012 (2)	−0.003 (2)	0.002 (2)
N13	0.0202 (18)	0.018 (2)	0.045 (4)	0.0087 (15)	0.004 (3)	0.012 (2)
O11	0.040 (4)	0.050 (5)	0.0295 (18)	0.023 (3)	0.007 (3)	0.004 (3)
O12	0.040 (2)	0.034 (2)	0.055 (4)	0.0256 (18)	0.018 (2)	0.010 (2)
O13	0.0310 (19)	0.0325 (17)	0.051 (4)	0.0180 (14)	−0.004 (2)	0.015 (2)
C11	0.026 (4)	0.020 (3)	0.024 (3)	0.010 (3)	0.008 (3)	0.010 (3)
C12	0.019 (2)	0.015 (2)	0.024 (4)	0.0092 (18)	0.004 (3)	0.002 (3)
C13	0.007 (3)	0.009 (3)	0.025 (2)	−0.002 (3)	0.0010 (18)	0.0077 (17)
C14	0.054 (6)	0.047 (7)	0.028 (6)	0.033 (5)	−0.004 (4)	0.016 (5)
C15	0.035 (4)	0.025 (2)	0.024 (3)	0.018 (3)	−0.005 (2)	0.0024 (18)
C16	0.042 (5)	0.047 (4)	0.024 (3)	0.028 (4)	0.002 (3)	0.011 (2)
N11A	0.033 (5)	0.027 (6)	0.027 (5)	0.011 (4)	0.007 (3)	0.014 (4)
N12A	0.020 (2)	0.0159 (18)	0.019 (3)	0.0107 (16)	0.0043 (19)	0.004 (2)
N13A	0.019 (2)	0.020 (2)	0.032 (3)	0.0085 (16)	0.006 (2)	0.012 (2)
O11A	0.031 (3)	0.036 (3)	0.0304 (18)	0.008 (2)	0.010 (3)	0.0074 (17)
O12A	0.041 (3)	0.049 (3)	0.050 (3)	0.035 (3)	0.030 (3)	0.032 (3)
O13A	0.034 (2)	0.0296 (15)	0.033 (3)	0.0163 (13)	−0.0055 (17)	0.0111 (16)
C11A	0.025 (4)	0.024 (3)	0.021 (3)	0.015 (3)	0.009 (3)	0.010 (3)
C12A	0.0166 (19)	0.018 (3)	0.016 (3)	0.0060 (19)	0.001 (2)	0.007 (3)
C13A	0.006 (3)	0.008 (3)	0.022 (2)	−0.004 (3)	0.0011 (17)	0.0072 (17)
C14A	0.057 (6)	0.042 (6)	0.023 (5)	0.045 (6)	0.018 (4)	0.009 (4)
C15A	0.024 (3)	0.0262 (19)	0.015 (2)	0.012 (2)	0.0007 (16)	0.0060 (14)
C16A	0.037 (4)	0.025 (2)	0.022 (2)	0.012 (3)	0.009 (3)	0.0104 (16)
N21	0.0342 (8)	0.0243 (7)	0.0263 (7)	0.0143 (6)	0.0021 (6)	0.0072 (6)
N22	0.0333 (8)	0.0331 (8)	0.0280 (7)	0.0192 (6)	0.0108 (6)	0.0207 (6)
N23	0.0287 (8)	0.0314 (9)	0.0875 (16)	0.0166 (7)	0.0213 (9)	0.0373 (10)
O21	0.0335 (7)	0.0498 (8)	0.0312 (7)	0.0258 (6)	0.0119 (5)	0.0185 (6)
O22	0.0565 (11)	0.0321 (9)	0.1166 (19)	0.0296 (9)	−0.0178 (12)	−0.0075 (10)
O23	0.1030 (17)	0.131 (2)	0.0963 (16)	0.1008 (17)	0.0702 (14)	0.0957 (17)
C21	0.0299 (9)	0.0222 (8)	0.0454 (11)	0.0116 (7)	0.0000 (8)	0.0007 (7)
C22	0.0243 (8)	0.0201 (7)	0.0522 (11)	0.0102 (6)	0.0080 (7)	0.0173 (8)
C23	0.0348 (9)	0.0254 (8)	0.0225 (7)	0.0163 (7)	0.0063 (6)	0.0135 (6)
C24	0.0541 (12)	0.0358 (10)	0.0248 (8)	0.0301 (9)	0.0044 (8)	0.0089 (7)
C25	0.0317 (9)	0.0647 (14)	0.0302 (9)	0.0243 (9)	0.0083 (7)	0.0295 (9)
C26	0.0383 (11)	0.0888 (18)	0.0413 (11)	0.0383 (12)	0.0188 (9)	0.0481 (13)
C1	0.0312 (11)	0.0628 (17)	0.0691 (18)	0.0079 (11)	0.0019 (11)	0.0160 (14)
O1	0.0709 (13)	0.0848 (15)	0.0653 (13)	0.0613 (13)	−0.0276 (11)	−0.0290 (11)
B1	0.0270 (9)	0.0233 (8)	0.0235 (8)	0.0066 (7)	0.0073 (7)	0.0053 (7)
F1	0.037 (3)	0.028 (2)	0.031 (2)	0.0183 (18)	0.0130 (18)	0.0090 (19)
F2	0.037 (3)	0.029 (2)	0.0255 (16)	0.015 (2)	0.0004 (15)	0.0061 (13)
F3	0.0250 (17)	0.0217 (15)	0.083 (5)	0.0004 (12)	0.007 (3)	0.007 (3)
F4	0.039 (2)	0.055 (3)	0.0244 (17)	0.0204 (19)	0.0125 (14)	0.0110 (17)
F1A	0.070 (4)	0.031 (2)	0.039 (3)	0.025 (2)	0.010 (2)	0.0146 (19)
F2A	0.0192 (14)	0.047 (3)	0.074 (4)	0.0064 (16)	−0.001 (2)	−0.015 (3)
F3A	0.0341 (16)	0.0302 (19)	0.068 (3)	0.0038 (13)	0.006 (2)	0.023 (2)
F4A	0.125 (6)	0.081 (4)	0.0201 (16)	0.033 (5)	0.019 (2)	0.008 (2)

### Geometric parameters (Å, º)

**Table:**

Ag—N11	2.082 (15)	C15A—C16A	1.510 (9)
Ag—N21	2.1193 (15)	C15A—H15C	0.9900
Ag—N11A	2.163 (16)	C15A—H15D	0.9900
N11—C13	1.325 (9)	C16A—H16C	0.9900
N11—C11	1.365 (9)	C16A—H16D	0.9900
N12—C13	1.356 (9)	N21—C23	1.338 (2)
N12—C12	1.389 (9)	N21—C21	1.365 (2)
N12—C15	1.467 (8)	N22—C23	1.350 (2)
N13—O12	1.224 (6)	N22—C22	1.380 (3)
N13—O13	1.233 (6)	N22—C25	1.466 (3)
N13—C12	1.436 (8)	N23—O23	1.212 (3)
O11—C16	1.431 (9)	N23—O22	1.221 (3)
O11—H11A	0.8400	N23—C22	1.429 (2)
C11—C12	1.345 (9)	O21—C26	1.413 (2)
C11—H11B	0.9500	O21—H21A	0.8400
C13—C14	1.479 (9)	C21—C22	1.349 (3)
C14—H14A	0.9800	C21—H21B	0.9500
C14—H14B	0.9800	C23—C24	1.486 (2)
C14—H14C	0.9800	C24—H24A	0.9800
C15—C16	1.509 (9)	C24—H24B	0.9800
C15—H15A	0.9900	C24—H24C	0.9800
C15—H15B	0.9900	C25—C26	1.527 (3)
C16—H16A	0.9900	C25—H25A	0.9900
C16—H16B	0.9900	C25—H25B	0.9900
N11A—C13A	1.365 (8)	C26—H26A	0.9900
N11A—C11A	1.379 (9)	C26—H26B	0.9900
N12A—C13A	1.369 (9)	C1—O1	1.410 (4)
N12A—C12A	1.389 (9)	C1—H1A	0.9800
N12A—C15A	1.483 (8)	C1—H1B	0.9800
N13A—O13A	1.226 (5)	C1—H1C	0.9800
N13A—O12A	1.231 (6)	O1—H1	0.8400
N13A—C12A	1.445 (8)	B1—F3	1.346 (6)
O11A—C16A	1.430 (9)	B1—F4A	1.366 (4)
O11A—H11C	0.8400	B1—F2A	1.373 (6)
C11A—C12A	1.355 (9)	B1—F4	1.381 (4)
C11A—H11D	0.9500	B1—F1A	1.382 (7)
C13A—C14A	1.491 (9)	B1—F1	1.395 (6)
C14A—H14D	0.9800	B1—F3A	1.399 (5)
C14A—H14E	0.9800	B1—F2	1.428 (6)
C14A—H14F	0.9800		
			
N11—Ag—N21	175.7 (5)	N12A—C15A—H15D	108.9
C13—N11—C11	111.5 (7)	C16A—C15A—H15D	108.9
C13—N11—Ag	128.4 (6)	H15C—C15A—H15D	107.7
C11—N11—Ag	121.4 (7)	O11A—C16A—C15A	112.9 (7)
C13—N12—C12	106.6 (7)	O11A—C16A—H16C	109.0
C13—N12—C15	122.1 (7)	C15A—C16A—H16C	109.0
C12—N12—C15	131.2 (7)	O11A—C16A—H16D	109.0
O12—N13—O13	125.0 (6)	C15A—C16A—H16D	109.0
O12—N13—C12	115.7 (5)	H16C—C16A—H16D	107.8
O13—N13—C12	119.2 (5)	C23—N21—C21	106.99 (16)
C16—O11—H11A	109.5	C23—N21—Ag	126.88 (12)
C12—C11—N11	105.1 (7)	C21—N21—Ag	126.14 (13)
C12—C11—H11B	127.5	C23—N22—C22	105.84 (15)
N11—C11—H11B	127.5	C23—N22—C25	124.53 (16)
C11—C12—N12	109.3 (7)	C22—N22—C25	129.62 (16)
C11—C12—N13	127.3 (6)	O23—N23—O22	123.6 (2)
N12—C12—N13	123.2 (6)	O23—N23—C22	119.0 (2)
N11—C13—N12	107.3 (7)	O22—N23—C22	117.3 (2)
N11—C13—C14	122.3 (8)	C26—O21—H21A	109.5
N12—C13—C14	127.2 (10)	C22—C21—N21	108.23 (17)
C13—C14—H14A	109.5	C22—C21—H21B	125.9
C13—C14—H14B	109.5	N21—C21—H21B	125.9
H14A—C14—H14B	109.5	C21—C22—N22	108.31 (15)
C13—C14—H14C	109.5	C21—C22—N23	126.3 (2)
H14A—C14—H14C	109.5	N22—C22—N23	125.40 (19)
H14B—C14—H14C	109.5	N21—C23—N22	110.63 (15)
N12—C15—C16	112.0 (8)	N21—C23—C24	124.38 (15)
N12—C15—H15A	109.2	N22—C23—C24	124.97 (16)
C16—C15—H15A	109.2	C23—C24—H24A	109.5
N12—C15—H15B	109.2	C23—C24—H24B	109.5
C16—C15—H15B	109.2	H24A—C24—H24B	109.5
H15A—C15—H15B	107.9	C23—C24—H24C	109.5
O11—C16—C15	112.1 (8)	H24A—C24—H24C	109.5
O11—C16—H16A	109.2	H24B—C24—H24C	109.5
C15—C16—H16A	109.2	N22—C25—C26	110.98 (19)
O11—C16—H16B	109.2	N22—C25—H25A	109.4
C15—C16—H16B	109.2	C26—C25—H25A	109.4
H16A—C16—H16B	107.9	N22—C25—H25B	109.4
C13A—N11A—C11A	103.4 (6)	C26—C25—H25B	109.4
C13A—N11A—Ag	122.6 (6)	H25A—C25—H25B	108.0
C11A—N11A—Ag	132.1 (8)	O21—C26—C25	111.84 (15)
C13A—N12A—C12A	104.6 (7)	O21—C26—H26A	109.2
C13A—N12A—C15A	127.3 (7)	C25—C26—H26A	109.2
C12A—N12A—C15A	127.5 (7)	O21—C26—H26B	109.2
O13A—N13A—O12A	125.3 (5)	C25—C26—H26B	109.2
O13A—N13A—C12A	118.6 (5)	H26A—C26—H26B	107.9
O12A—N13A—C12A	116.1 (5)	O1—C1—H1A	109.5
C16A—O11A—H11C	109.5	O1—C1—H1B	109.5
C12A—C11A—N11A	110.0 (10)	H1A—C1—H1B	109.5
C12A—C11A—H11D	124.5	O1—C1—H1C	109.5
N11A—C11A—H11D	124.5	H1A—C1—H1C	109.5
C11A—C12A—N12A	107.9 (7)	H1B—C1—H1C	109.5
C11A—C12A—N13A	125.8 (7)	C1—O1—H1	109.5
N12A—C12A—N13A	126.2 (7)	F4A—B1—F2A	110.2 (4)
N11A—C13A—N12A	112.9 (7)	F3—B1—F4	112.8 (4)
N11A—C13A—C14A	123.6 (8)	F4A—B1—F1A	110.7 (5)
N12A—C13A—C14A	122.9 (9)	F2A—B1—F1A	110.7 (5)
C13A—C14A—H14D	109.5	F3—B1—F1	111.9 (5)
C13A—C14A—H14E	109.5	F4—B1—F1	109.9 (6)
H14D—C14A—H14E	109.5	F4A—B1—F3A	108.5 (4)
C13A—C14A—H14F	109.5	F2A—B1—F3A	108.4 (5)
H14D—C14A—H14F	109.5	F1A—B1—F3A	108.4 (5)
H14E—C14A—H14F	109.5	F3—B1—F2	107.7 (5)
N12A—C15A—C16A	113.4 (8)	F4—B1—F2	107.7 (3)
N12A—C15A—H15C	108.9	F1—B1—F2	106.5 (5)
C16A—C15A—H15C	108.9		
			
C13—N11—C11—C12	1.2 (14)	Ag—N11A—C13A—N12A	−178.7 (9)
Ag—N11—C11—C12	−179.8 (8)	C11A—N11A—C13A—C14A	166.5 (12)
N11—C11—C12—N12	1.2 (14)	Ag—N11A—C13A—C14A	−12 (2)
N11—C11—C12—N13	−173.5 (11)	C12A—N12A—C13A—N11A	−4.3 (15)
C13—N12—C12—C11	−3.1 (15)	C15A—N12A—C13A—N11A	−176.3 (12)
C15—N12—C12—C11	175.3 (13)	C12A—N12A—C13A—C14A	−168.1 (12)
C13—N12—C12—N13	171.8 (10)	C15A—N12A—C13A—C14A	20 (2)
C15—N12—C12—N13	−10 (2)	C13A—N12A—C15A—C16A	93.2 (15)
O12—N13—C12—C11	−10.3 (15)	C12A—N12A—C15A—C16A	−77.0 (15)
O13—N13—C12—C11	168.2 (11)	N12A—C15A—C16A—O11A	−71.9 (10)
O12—N13—C12—N12	175.6 (11)	C23—N21—C21—C22	−0.5 (2)
O13—N13—C12—N12	−5.8 (16)	Ag—N21—C21—C22	179.02 (13)
C11—N11—C13—N12	−3.2 (14)	N21—C21—C22—N22	0.6 (2)
Ag—N11—C13—N12	178.0 (9)	N21—C21—C22—N23	−179.57 (17)
C11—N11—C13—C14	−168.5 (12)	C23—N22—C22—C21	−0.4 (2)
Ag—N11—C13—C14	10 (2)	C25—N22—C22—C21	−179.75 (19)
C12—N12—C13—N11	3.8 (14)	C23—N22—C22—N23	179.73 (17)
C15—N12—C13—N11	−174.8 (11)	C25—N22—C22—N23	0.4 (3)
C12—N12—C13—C14	167.9 (13)	O23—N23—C22—C21	168.6 (2)
C15—N12—C13—C14	−11 (2)	O22—N23—C22—C21	−10.3 (3)
C13—N12—C15—C16	103.4 (13)	O23—N23—C22—N22	−11.6 (3)
C12—N12—C15—C16	−74.8 (16)	O22—N23—C22—N22	169.5 (2)
N12—C15—C16—O11	−68.9 (10)	C21—N21—C23—N22	0.2 (2)
C13A—N11A—C11A—C12A	1.5 (18)	Ag—N21—C23—N22	−179.28 (12)
Ag—N11A—C11A—C12A	−179.9 (13)	C21—N21—C23—C24	−178.24 (19)
N11A—C11A—C12A—N12A	−2.1 (15)	Ag—N21—C23—C24	2.2 (3)
N11A—C11A—C12A—N13A	−179.3 (10)	C22—N22—C23—N21	0.1 (2)
C13A—N12A—C12A—C11A	3.7 (15)	C25—N22—C23—N21	179.47 (17)
C15A—N12A—C12A—C11A	175.8 (12)	C22—N22—C23—C24	178.57 (18)
C13A—N12A—C12A—N13A	−179.0 (10)	C25—N22—C23—C24	−2.1 (3)
C15A—N12A—C12A—N13A	−7 (2)	C23—N22—C25—C26	−96.7 (2)
O13A—N13A—C12A—C11A	176.7 (10)	C22—N22—C25—C26	82.5 (2)
O12A—N13A—C12A—C11A	−2.9 (15)	N22—C25—C26—O21	52.0 (3)
O13A—N13A—C12A—N12A	−0.1 (16)	C13—N11—N21—C23	−160.8 (9)
O12A—N13A—C12A—N12A	−179.7 (11)	C13A—N11A—N21—C23	−148.6 (11)
C11A—N11A—C13A—N12A	0.8 (18)		

### Hydrogen-bond geometry (Å, º)

**Table:**

*D*—H···*A*	*D*—H	H···*A*	*D*···*A*	*D*—H···*A*
O1—H1···F2	0.84	1.84	2.673 (9)	173
O11—H11*A*···O1	0.84	1.86	2.697 (10)	175
O21—H21*A*···O11^i^	0.84	1.91	2.726 (11)	164
O21—H21*A*···O11*A*^i^	0.84	1.87	2.712 (11)	176

Symmetry code: (i) *x*, *y*, *z*−1.
